# Artificial Intelligence in the Diagnosis of Pediatric Rare Diseases: From Real-World Data Toward a Personalized Medicine Approach

**DOI:** 10.3390/jpm15090407

**Published:** 2025-09-01

**Authors:** Nikola Ilić, Adrijan Sarajlija

**Affiliations:** 1 Clinical Genetics Outpatient Clinic, Mother and Child Health Care Institute of Serbia “Dr. Vukan Cupic”, 11070 Belgrade, Serbia; adrijan.sarajlija@imd.org.rs; 2 Department of Pediatrics, Faculty of Medicine, University of Belgrade, 11000 Belgrade, Serbia; 3 Faculty of Medicine, University of Eastern Sarajevo, 73300 Foča, Bosnia and Herzegovina

**Keywords:** artificial intelligence (AI), pediatric rare diseases, genomic diagnostics, personalized medicine, large language models (LLMs), real-world data, ethical considerations

## Abstract

**Background:** Artificial intelligence (AI) is increasingly applied in the diagnosis of pediatric rare diseases, enhancing the speed, accuracy, and accessibility of genetic interpretation. These advances support the ongoing shift toward personalized medicine in clinical genetics. Objective: This review examines current applications of AI in pediatric rare disease diagnostics, with a particular focus on real-world data integration and implications for individualized care. **Methods:** A narrative review was conducted covering AI tools for variant prioritization, phenotype–genotype correlations, large language models (LLMs), and ethical considerations. The literature was identified through PubMed, Scopus, and Web of Science up to July 2025, with priority given to studies published in the last seven years. **Results:** AI platforms provide support for genomic interpretation, particularly within structured diagnostic workflows. Tools integrating Human Phenotype Ontology (HPO)-based inputs and LLMs facilitate phenotype matching and enable reverse phenotyping. The use of real-world data enhances the applicability of AI in complex and heterogeneous clinical scenarios. However, major challenges persist, including data standardization, model interpretability, workflow integration, and algorithmic bias. **Conclusions:** AI has the potential to advance earlier and more personalized diagnostics for children with rare diseases. Achieving this requires multidisciplinary collaboration and careful attention to clinical, technical, and ethical considerations.

## 1. Introduction

Rare diseases affect millions of children worldwide. Although each individual condition is uncommon, together they form a significant clinical and public health challenge. For many families, the path to diagnosis, often referred to as the diagnostic odyssey, is long, uncertain, and emotionally draining [1]. According to a 2017 EURORDIS survey, the average diagnostic journey for patients with rare diseases spans 5–7 years, often involving multiple misdiagnoses and fragmented care [2]. In pediatric practice, rare diseases frequently manifest as unexplained developmental delays, multisystem anomalies, or atypical syndromes. Examples include genetic syndromes such as Noonan syndrome, skeletal dysplasias like achondroplasia, and metabolic disorders such as phenylketonuria. These conditions illustrate the diversity of rare disease presentations, from recognizable dysmorphic patterns to subtle biochemical abnormalities. Over 70% have a genetic basis, and most begin in early childhood. Timely and accurate diagnosis is essential not only for therapeutic decisions but also for genetic counseling, family planning, and prognosis [3].

Yet, even with the widespread availability of next-generation sequencing technologies, such as whole-exome (WES) and whole-genome sequencing (WGS), interpreting the massive volume of genetic data remains a critical bottleneck. The shortage of trained clinical geneticists, variability in phenotypic documentation, and fragmented health information systems further delay diagnosis or lead to missed opportunities. Additional barriers include limited interoperability between genomic databases and hospital EMRs, variability in variant interpretation standards between laboratories, and the absence of unified guidelines for integrating genomic and clinical data. Collectively, these challenges contribute to prolonged diagnostic odysseys and underscore the need for tools that can operate effectively in heterogeneous, resource-variable environments. They are especially pronounced in low-resource settings or outside tertiary centers [4].

In response, artificial intelligence (AI) is increasingly being explored as a potentially valuable tool to support rare disease diagnostics. AI in medicine commonly encompasses machine learning (ML), artificial neural networks, and deep learning approaches that learn patterns from clinical data to support tasks such as image interpretation, signal analysis, electronic health record phenotyping, and clinical decision support. These paradigms have matured rapidly and now underpin many practical applications across medical specialties [5].

From variant interpretation to phenotype–genotype correlations, modern AI systems have been explored as tools that can help prioritize findings, support differential diagnosis, and potentially reduce clinicians’ cognitive burden. Their scalability and adaptability may render them valuable both in high-resource institutions and in settings with limited specialist access [6].

This review explores the current landscape of AI applications in the diagnosis of pediatric rare diseases, with a main focus on their integration into personalized medicine. We highlight recent comparative evidence in the diagnostic performance of AI vs. human experts—both from structured studies and real-world clinical applications. In doing so, we examine not only the theoretical potential of AI in genomic interpretation and phenotype–genotype correlations but also how these tools function under real-world conditions. Furthermore, we discuss the practical, ethical, and technical challenges associated with implementing AI technologies in pediatric care, particularly in settings where infrastructure, training, and data standardization remain variable.

Ultimately, we aim to explore how AI can serve not as a replacement but as a valuable ally in achieving faster, more accurate, and personalized care for children with rare conditions.

## 2. Materials and Methods

This narrative review was conducted to synthesize current knowledge on the application of artificial intelligence (AI) in the diagnosis of pediatric rare diseases.

### 2.1. Research Question

The guiding research question was as follows: What are the current applications, opportunities, and challenges of AI in the diagnosis of pediatric rare diseases, particularly regarding real-world data integration and implications for personalized medicine?

### 2.2. Search Strategy

A literature search was performed in PubMed, Scopus, and Web of Science from database inception to July 2025. The following search string was used, with slight modifications for each database:

(“artificial intelligence” OR “machine learning” OR “deep learning” OR “large language model”) AND (“rare disease*” OR “genetic disorder*”) AND (“child*” OR “pediatric*”)*.

### 2.3. Inclusion Criteria

– Peer-reviewed original research articles, systematic reviews, meta-analyses, and narrative reviews.
– Publications directly addressing AI applications in pediatric rare disease diagnostics, including variant interpretation, phenotype–genotype correlations, clinical decision support, or large language models.
– Articles published in English.

### 2.4. Exclusion Criteria

– Studies focusing exclusively on adult populations.
– Purely technical AI model development papers without clinical or diagnostic relevance.
– Conference abstracts, editorials, and commentaries without peer-reviewed full text.

### 2.5. Study Selection

The initial search retrieved approximately 180 records (PubMed = 70, Scopus = 60, and Web of Science = 50). After screening the titles and abstracts and applying inclusion and exclusion criteria, 55 studies were retained for full-text review and narrative synthesis. Additional references were identified through manual searches of the cited literature in key publications.

This structured approach was designed to minimize selection bias while allowing a comprehensive synthesis of the most relevant and clinically meaningful literature.

## 3. Artificial Intelligence in the Diagnosis of Pediatric Rare Diseases

### 3.1. The Role of AI in Genomic Data Interpretation

The advent of NGS has substantially transformed the diagnostic landscape of genetic medicine, particularly in the field of rare diseases. By enabling high-throughput parallel sequencing, NGS allows the simultaneous analysis of hundreds or thousands of genes. Consequently, NGS has greatly enhanced diagnostic efficiency and helped expand the horizons of personalized medicine. Diagnostic yields have increased significantly, with reported rates ranging from 40% to over 70%, depending on the specific patient cohort and clinical context. As a result, gene panels and whole-exome sequencing (WES) have become the standard of care in many tertiary centers worldwide [7,8].

Crucially, NGS has shifted the diagnostic paradigm from a traditional linear phenotype-to-genotype workflow toward a more integrated and bidirectional approach. Molecular findings can help to refine or even redefine clinical hypotheses, and the clinical context shapes the interpretation of genetic data. This process, often termed reverse phenotyping (RF), has proven particularly valuable in rare diseases characterized by atypical, evolving, or overlapping features where classical syndromic recognition may fall short of establishing a definitive diagnosis [9].

However, despite its potential clinical utility, NGS is not without limitations. The technology frequently generates variants of uncertain significance (VUSs), uncovers incidental or secondary findings, and may detect mutations in genes not previously linked to the suspected phenotype. Furthermore, certain categories of pathogenic alterations (including deep intronic variants, structural rearrangements, epimutations, and complex copy number variants) may be missed by exome-based approaches. This brings the necessity for the application of whole-genome sequencing (WGS) for more comprehensive coverage. Yet, the clinical implementation of WGS introduces additional layers of complexity, including data interpretation challenges and ethical considerations [10,11].

Importantly, even when a pathogenic or likely pathogenic variant is identified, assigning the correct diagnostic label remains a demanding task. Accurate interpretation requires molecular confirmation integrated with expert assessment of the patient’s clinical features, laboratory data, imaging studies, and global developmental trajectory or familial context. This is especially challenging in pediatric populations, where phenotypic expression may be incomplete or age-dependent, making classical diagnostic patterns harder to recognize [9,12].

Given these complex circumstances, multidisciplinary collaboration has become the cornerstone of rare disease diagnostics. Optimal interpretation requires close cooperation between clinical geneticists, subspecialty clinicians, molecular biologists, radiologists, and genetic counselors to contextualize genomic findings within the broader clinical picture. However, this model is resource-intensive and not always accessible in under-resourced settings [12].

#### 3.1.1. AI Tools for Variant Interpretation

In response to these challenges, there is a growing effort for the development of advanced decision-support tools capable of assisting clinicians in the complex task of genomic interpretation [13] (Table 1).

Modern AI platforms can automate variant prioritization by integrating pathogenicity predictions, population frequency data, inheritance patterns, and gene–disease associations in real time. For example, tools such as MOON (by Diploid), Fabric Genomics, Emedgene, and GEM utilize phenotype-driven algorithms that match Human Phenotype Ontology (HPO) terms to known gene–disease relationships. This process can effectively narrow down candidate variants that require further clinician review [14].

#### 3.1.2. Role of Large Language Models (LLMs)

A potentially promising subset of AI applications involves large language models (LLMs), which are designed to process natural language input and generate human-like responses. Preliminary studies suggest that models such as OpenAI’s ChatGPT and DeepSeek may have the capacity to approximate certain aspects of diagnostic reasoning and to propose possible differential diagnoses when supplied with detailed clinical input [6].

In the context of pediatric rare disease diagnostics, large language models (LLMs) may support hypothesis generation, contribute to the reinterpretation of existing genomic data, and suggest potential genetic conditions even when provided with limited input. Early studies and anecdotal institutional experiences have begun to examine their possible roles in reverse phenotyping, particularly in complex or ambiguous cases where conventional phenotype-driven workflows have not provided actionable leads [15].

However, their clinical utility remains largely experimental. Unlike dedicated variant interpretation tools, LLMs are not yet optimized for structured genomic inputs and lack formal integration into diagnostic pipelines [6]. A key concern is the phenomenon of hallucination—where models produce outputs that are syntactically valid but factually incorrect. This has been extensively documented in recent evaluations of generative AI tools in medical and non-medical domains [16]. This is particularly problematic in rare disease diagnostics, where subtle nuances determine clinical decisions. Moreover, LLMs may reflect biases present in their training data, leading to an overrepresentation of well-documented conditions and underrepresentation of phenotypic variability. These concerns mirror well-documented disparities observed in algorithmic performance when trained on demographically skewed datasets [17,18].

The lack of transparency in model architecture and training data further complicates their validation. Without rigorous benchmarking against expert consensus and real-world data, LLMs cannot yet be considered reliable standalone tools. Nevertheless, their potential to complement expert-driven interpretation is significant, particularly as user interfaces evolve and integration with electronic health records becomes feasible [19].

Furthermore, critical questions remain regarding how AI outputs compare to multidisciplinary human expert judgment, especially in a rare disease diagnostic scenario. Rigorous benchmarking studies are needed to evaluate AI models against both each other and established clinical standards in realistic, patient-centered contexts [20].

Ultimately, LLMs represent a novel and dynamic frontier in AI-assisted medicine, but their integration into pediatric rare disease diagnostics demands caution, validation, and continual human oversight [21].

### 3.2. Phenotype–Genotype Integration Through Automated Tools

The diagnostic yield of genomic sequencing is strongly influenced by the quality, completeness, and granularity of phenotypic data provided alongside molecular analyses. In the field of pediatric rare disease diagnostics, clinical presentations are frequently complex, evolving, or syndromically overlapping. This makes accurate phenotyping as critical as the sequencing itself. Unlike adult-onset conditions, many pediatric disorders display age-dependent features, incomplete penetrance, or subtle morphological signs that may initially escape recognition. This diagnostic complexity often results in delayed or missed diagnoses, especially for rare conditions with variable expressivity [22].

Traditional approaches to phenotyping rely heavily on the clinician’s expertise to recognize distinctive patterns and manually match them to known disorders. While experienced dysmorphologists and geneticists can achieve impressive diagnostic accuracy, this process is inherently subjective and variable. It depends not only on individual knowledge but also on precise clinical measurements, standardized terminology, and systematic recording of subtle physical or developmental features. Small inconsistencies, such as imprecise anthropometric data, incomplete family history, or missing seemingly minor anomalies, can significantly alter diagnostic pathways and interpretations [23].

AI-driven platforms have provided important advances by enabling structured, scalable, and automated approaches to determine phenotype–genotype correlations. Central to this development is the widespread adoption of the Human Phenotype Ontology (HPO), which standardizes clinical features into a hierarchical and computable terminology. By encoding patient phenotypes using HPO terms, clinicians can now input structured phenotypic profiles into AI tools, facilitating computational matching with curated disease databases [24,25].

Once phenotypic data are encoded, AI platforms apply algorithms that prioritize candidate genes and associated disorders based on semantic similarity, probabilistic modeling, and evidence-based knowledge graphs. This approach minimizes the biases and inconsistencies inherent in purely manual interpretation while maximizing diagnostic spread, especially for conditions with non-classical or overlapping phenotypes [26].

A growing array of tools exemplifies this integration: Phenomizer calculates statistical matches between patient phenotype sets and known Mendelian conditions, enabling rapid narrowing of diagnostic possibilities based on similarity scoring. GEM (Genetic Evaluation Module) combines phenotype scoring with a variant pathogenicity analysis to generate ranked, integrated diagnostic hypotheses. Face2Gene, leveraging deep learning and facial recognition algorithms, analyzes facial morphology to suggest syndromic diagnoses, effectively serving as a digital dysmorphologist that supports clinician assessments. AMELIE mines the scientific literature to prioritize candidate genes based on both phenotypic features and gene relevance, bridging genomic data with up-to-date knowledge in the medical literature [27,28] (Table 2).

AI tools have, in many cases, demonstrated advantages over traditional keyword searches or manual OMIM queries, with the evidence indicating a reduced diagnostic time and expanded support for clinicians’ decision-making. They are particularly valuable in resource-constrained settings, regional centers without subspecialty expertise, or scenarios where rapid diagnostic triage is essential [29].

An additional and rapidly evolving layer of genotype–phenotype integration involves LLMs. Unlike structured phenotype-driven algorithms alone, LLMs can flexibly interpret natural language descriptions of symptoms, examination findings, and historical narratives [30] (Table 3).

They can further map them to HPO-like representations or relevant diagnostic concepts. This allows AI to function effectively even when phenotypic data are unstructured, incomplete, or non-standardized, which is a frequent reality in everyday pediatric clinical practice [17].

Moreover, by integrating LLM capabilities with existing phenotype–genotype tools, AI systems can help synthesize diverse information streams. Structured HPO terms, free-text clinical notes, imaging descriptions, and literature evidence can all be transformed into coherent, ranked diagnostic suggestions. This holistic approach has been associated with improvements in diagnostic efficiency and accuracy, with the potential to reduce clinicians’ workload and lower the risk of missed rare diagnoses [20].

Ultimately, the automation and augmentation of phenotype–genotype correlations through AI-driven tools reinforce the foundation of personalized medicine in pediatric genetics. These tools are empowering clinicians to move beyond the limitations of human memory or single-specialist expertise. This approach offers open access to advanced diagnostic insights and paves the way toward faster, more accurate, and equitable care for children with rare diseases [25].

### 3.3. Real-World Data: Opportunities and Challenges for AI-Assisted Rare Disease Diagnosis

The diagnostic journey for children with rare diseases is often complex, prolonged, and nonlinear. For many families, it involves years of uncertainty, repeated hospital visitations, and inconclusive investigations. This process is known as the “diagnostic odyssey” [1]. Rare diseases, by their nature, frequently defy the rigid frameworks of randomized controlled trials (RCTs). While RCTs remain the gold standard for evaluating therapeutic interventions, they are often impractical or unsuitable for answering diagnostic questions in the rare disease setting [31].

In this context, real-world data (RWD) has emerged as an invaluable source of insight. Unlike RCTs, which depend on strict inclusion criteria, controlled environments, and predefined outcomes, RWD reflects the authentic complexity of clinical practice. It is longitudinal, multimodal, and captures how diseases manifest, progress, and respond to interventions in actual patients, not idealized study populations. RWD is generated from diverse sources, including electronic health records (EHRs), patient registries, diagnostic and genomic databases, administrative health records, and, increasingly, from digital health tools such as mobile applications and wearable devices [32].

Importantly, RWD encompasses a broader spectrum of clinical variability. This corpus of data includes patients that are often excluded from clinical trials due to comorbidities, atypical presentations, or age restrictions. This makes it especially valuable for understanding the full phenotypic spectrum of rare diseases, capturing early signs, variable expressivity, and real-life treatment responses across diverse pediatric populations [33].

However, the organic nature of RWD presents significant challenges. These data are often unstructured and heterogeneous. They are stored in free-text clinical notes, radiology reports, laboratory systems, or fragmented across institutional archives. Terminology may be inconsistent, documentation incomplete, and phenotypic descriptions scattered without standardized frameworks. Genetic findings are frequently stored separately from phenotypic data, limiting integrated analysis. Consequently, the analytical utility of RWD is often underexploited [34].

AI is uniquely positioned to overcome these limitations. Modern AI tools, including natural language processing (NLP) and LLMs, can automatically extract structured information from unstructured text, map clinical observations to standardized terminologies such as Human Phenotype Ontology (HPO) terms, and harmonize data across disconnected datasets. Beyond extraction, AI systems can impute missing information, identify patterns across time, cluster patients based on phenotypic similarity, and detect rare associations that may elude human review [30].

AI models have the potential to learn from new patient data through adaptive feedback loops, which may help refine diagnostic hypotheses and improve performance over time. In contrast to many traditional statistical methods that typically require clean, complete datasets and can be highly sensitive to missing or inconsistent information, AI approaches have demonstrated a greater tolerance for such imperfect conditions. This feature is particularly relevant in rare disease contexts, where large, homogeneous, and standardized cohorts are seldom available. In these circumstances, even individual cases—despite incomplete data—may incrementally contribute to the broader accumulation of clinical insights [25].

Despite this promise, transforming RWD into structured, operable, and analytically usable databases presents significant challenges. Data interoperability remains a major barrier, with differing standards and architectures across healthcare systems limiting integration. Privacy concerns, especially for pediatric populations, add an essential ethical dimension requiring robust governance frameworks. Moreover, trust in AI-generated outputs must be carefully built. If models are trained on non-representative datasets, there is a risk of bias propagation, leading to unequal diagnostic accuracy across demographic groups [35].

To fully harness RWD’s potential in rare disease diagnostics, a multilayered infrastructure is needed. This includes standardized data collection protocols, harmonized phenotype–genotype registries, validation studies, and transparent AI pipelines that integrate seamlessly into clinical workflows. Within this ecosystem, AI should not be viewed merely as an analytical tool but rather as an active partner across the data lifecycle—from acquisition and curation to analysis, interpretation, and clinical application [36].

By combining the scalability and pattern-recognition capabilities of AI with the authenticity and richness of real-world data, a new diagnostic paradigm can emerge in pediatric genetics—one that does not begin in idealized study settings, but is rooted in the day-to-day complexity of medicine as practiced. In this paradigm, AI can serve as an important tool for translating real-world evidence into potential diagnostic value, supporting faster identification of rare diseases, assisting in earlier intervention, and contributing to more personalized and potentially more equitable care for children worldwide [34].

### 3.4. Comparative Diagnostic Performance of AI and Human Experts

Despite the growing momentum of AI technologies in pediatric rare disease diagnostics, several barriers continue to limit their broad and seamless adoption into clinical practice. These challenges extend beyond technical limitations and involve issues related to data quality, clinical workflows, and regulatory uncertainty [25].

This section focuses specifically on the performance-related limitations of AI tools in rare disease diagnostics. Here, we highlight challenges that emerge directly from algorithmic evaluation, such as bias revealed during benchmarking, overgeneralization across heterogeneous patient cohorts, and weaknesses identified when comparing AI systems to human experts. To provide a more structured overview, Table 4 categorizes these obstacles based on their origin, highlights how they affect diagnostic effectiveness, and suggests potential mitigation strategies.

Several recent studies have sought to address this by benchmarking AI-assisted diagnostics against expert-led evaluations using real-world clinical cases of rare diseases. Controlled investigations involving genetically confirmed disorders suggest that AI platforms, ranging from phenotype-driven algorithms such as GEM to LLMs like ChatGPT and DeepSeek Medical AI, exhibit moderate to high diagnostic accuracy under specific conditions. However, their performance is highly variable and context-dependent, influenced by factors such as the disease type, phenotypic clarity, data structure, and the complexity of individual cases [21,37,38].

For instance, AI systems have shown promising performance in diagnosing some common and syndromically well-defined conditions, such as achondroplasia, osteogenesis imperfecta, or Noonan syndrome. These disorders are often characterized by distinctive, recognizable phenotypic features that align relatively closely with curated training data and established genotype–phenotype associations represented in AI knowledge bases. In such contexts, AI tools may be able to match phenotypic inputs to known diagnostic entities, suggesting possible diagnoses with limited human input and, in certain cases, generating results within a short timeframe [39].

However, AI performance declines notably when faced with ultra-rare, genetically heterogeneous, or clinically ambiguous conditions. In these scenarios, real-life clinical judgment becomes essential. Diagnostic accuracy depends not only on recognizing textbook features, but also on integrating subtle, context-specific clues and elements that may not be explicitly encoded within AI training datasets. Additionally, ultra-rare diseases, by definition, lack large-scale representation in public databases, limiting AI’s ability to draw upon prior examples for pattern recognition [39].

One comparative study evaluated AI models and human experts using a dataset of pediatric rare bone disease cases with confirmed molecular diagnoses. Experienced human clinicians achieved diagnostic accuracies exceeding 80%, reflecting the depth of their specialized training, clinical reasoning, and capacity to synthesize disparate data points into coherent diagnostic hypotheses. In contrast, AI models in the same study achieved accuracies in the range of 60–65%, depending on the complexity of cases, formulation of the input data, and the type of AI system employed [40].

Interestingly, combined approaches, where outputs from multiple AI tools were integrated, often resulted in improved diagnostic yield. This finding highlights the potential of multi-model strategies, analogous to multidisciplinary clinical discussions where diverse perspectives converge to refine a diagnosis. Such combined AI models can leverage complementary strengths of different systems, combining deep learning models’ semantic reasoning with phenotype-driven tools’ structured ontology matching to achieve higher accuracy [41].

Importantly, when AI-generated diagnoses were correct, their reasoning pathways demonstrated high concordance with human diagnostic logic. Models produced overlapping differential diagnoses, prioritized similar key phenotypic features, and aligned with expert interpretations. This convergence suggests that AI systems, when provided with sufficient structured data, can mirror aspects of human diagnostic reasoning, particularly for straightforward or classic presentations [42].

In comparing AI-based diagnostic outputs with those of human experts, it is important to consider not only accuracy but also confidence levels associated with each decision. Previous studies have shown that while AI models often achieve comparable or even higher diagnostic accuracy in certain tasks, their confidence levels can vary widely depending on the complexity of the case and the specificity of training data. Similarly, human experts tend to exhibit calibrated self-assessment, generally showing higher confidence in correct diagnoses and lower confidence when uncertain. In contrast, AI systems may demonstrate either overconfidence or underconfidence due to limitations in probabilistic calibration [40].

However, AI models remain susceptible to certain limitations. Overgeneralization is a recurrent challenge, with models occasionally proposing broad syndromic categories without sufficiently discriminating among closely related entities. Misinterpretation of delicate phenotypic cues, such as subtle dysmorphisms, behavioral features, or growth patterns, also limits diagnostic precision, especially when inputs are incomplete, unstructured, or described in non-standardized language. Additionally, AI lacks the ability to contextualize findings within psychosocial, cultural, or family-specific frameworks, aspects that are often critical to holistic clinical assessments [19].

Despite these limitations, AI models consistently deliver faster turnaround times compared to traditional diagnostic workflows. They can process vast datasets within seconds to minutes, perform unbiased variant assessments, and generate reproducible outputs without fatigue or cognitive bias. This scalability makes them invaluable in triaging large numbers of cases, flagging potentially actionable findings, and generating early diagnostic hypotheses that guide subsequent expert review and targeted testing strategies [43].

Beyond efficiency, AI systems contribute to democratize the availability of the expertise. In settings where access to experienced clinical geneticists is limited, AI tools can serve as surrogate diagnostic aids, elevating the diagnostic capacity of the physicians. This holds particular promise in under-resourced regions, where the shortage of trained personnel remains a significant barrier to a timely rare disease diagnosis [44].

### 3.5. Challenges in Clinical Implementation

In contrast to the performance-level concerns outlined above, this section is dedicated to the pragmatic and systemic barriers encountered in clinical implementation. Several interrelated obstacles must be addressed before AI tools can be fully embedded into everyday pediatric care. These can be grouped into three main categories: data-related issues, workflow and clinician adoption, and regulatory or ethical barriers (Table 5) [41].

AI platforms require access to high-quality, structured data, but many healthcare systems still rely on unstructured electronic medical records (EMRs). Phenotypic descriptions are often buried in free-text notes, inconsistently documented, or incomplete, which limits the functionality of AI tools dependent on standardized inputs such as HPO terms. Data fragmentation across institutions and the lack of interoperability between EMRs and genomic databases further hampers integrated analysis and AI-assisted interpretation. Automated natural language processing (NLP) could mitigate this issue, but its clinical implementation and validation are still limited.

One of the most pressing challenges is data interoperability. For AI platforms to function optimally, they require access to structured, high-quality clinical and genomic data. However, many healthcare systems continue to rely on electronic medical records (EMRs) that are predominantly unstructured, with phenotypic information buried within free-text clinical notes. Documentation practices vary significantly across clinicians, specialties, and institutions, resulting in inconsistent terminology and incomplete phenotypic capture. This heterogeneity severely limits the utility of phenotype-driven AI tools, which depend on standardized inputs such as HPO terms to generate accurate diagnostic suggestions [45].

Automated natural language processing systems, which can extract and map free-text descriptions to structured ontologies, are still relatively underutilized in clinical genomics. Their broader integration into routine workflows has the potential to substantially improve data standardization and accessibility. However, challenges related to technical implementation, validation, and clinician trust in automated data extraction remain ongoing areas of development [46].

Another major challenge is the lack of standardization across AI platforms. Different tools employ distinct algorithms, knowledge bases, variant classification frameworks, and decision-support logic. This variability leads to discrepancies in outputs, with potentially conflicting diagnostic suggestions for the same patient. In the absence of universally accepted guidelines or consensus statements outlining which AI tools to use, clinicians may hesitate to rely on them in high-stakes diagnostic decisions [41].

The integration of AI tools into existing clinical workflows poses significant barriers. Many current solutions function as standalone platforms that require separate logins, cumbersome data uploads, and manual retrieval of outputs. This digital fragmentation disrupts diagnostic pipelines and adds operational burdens to already overstretched clinical teams. True clinical utility demands seamless interoperability between AI systems, sequencing laboratories, electronic medical records (EMRs), and hospital information systems, enabling streamlined data flow without duplication of effort. However, technical limitations, such as heterogeneous EHR architectures, legacy infrastructures, and inconsistent implementation of standards like HL7 FHIR, still prevent full integration and hinder adoption [47].

Beyond technical barriers, organizational and human factors complicate integration. Resistance to change, lack of formal training, unclear accountability, and misalignment of incentives can stall uptake. Successful integration requires co-design with clinicians, robust governance, and alignment with clinical needs and regulatory frameworks [48].

Moreover, the need for human oversight introduces a paradoxical complexity. While AI systems can accelerate variant interpretation and phenotype matching, their outputs still require clinical validation and contextual judgment. Ultimately, the responsibility for diagnostic decisions remains with physicians, who must critically assess AI-generated recommendations before acting upon them. Without thoughtful implementation, this dual-layer model can inadvertently increase clinicians’ workload rather than alleviate it, creating additional cognitive and operational burdens [49].

Training and trust represent other significant challenges. Many clinicians remain unfamiliar with AI methodologies, underlying model architectures, and potential limitations. The perception of AI as a “black box” system with unclear reasoning pathways fuels skepticism and reluctance to fully integrate its outputs into clinical decision-making. Targeted education on AI fundamentals, strengths, and weaknesses, as well as exposure to its practical use, is essential for the meaningful and informed adoption and avoidance of superficial or misapplied utilization [50].

In addition to clinician-focused education, regulatory and governance frameworks are beginning to directly address the risks of opaque or “black box” AI models. The EU AI Act (2024) now classifies medical AI as a high-risk application, requiring transparency, documentation, and risk monitoring; international standards such as ISO/IEC 23894:2023 [51] and WHO guidance likewise emphasize explainability and accountability in clinical AI deployment. These frameworks aim to complement educational efforts by embedding transparency and oversight requirements into system design and hospital integration [52].

Finally, regulatory frameworks have yet to keep pace with the speed of AI innovation. Questions surrounding accountability, liability, data governance, and validation standards for AI-driven medical recommendations remain largely unresolved. This is particularly critical in pediatrics, where the legal and ethical dimensions of diagnostic decisions are much more challenging given the patient vulnerability, parental consent dynamics, and the potential long-term impact of diagnostic labeling on a child’s life trajectory. Ensuring that AI models are trained on diverse and representative pediatric datasets is essential to avoid propagating biases that could exacerbate existing health disparities [44].

Additionally, concerns around data privacy and security are amplified in the context of genomic and phenotypic data, which are easily identifiable. Robust safeguards are necessary to maintain public trust and comply with evolving legal frameworks, such as national data protection regulations [53].

Addressing these complex challenges will require a coordinated, multidisciplinary effort. Developers, clinicians, bioinformaticians, hospital administrators, ethicists, and policymakers must collaboratively design implementation strategies. These strategies must prioritize technical robustness, clinical relevance, ethical integrity, and operational feasibility. Transparent model validation, explainability of AI outputs, user-friendly interfaces embedded within clinical workflows, and ongoing post-implementation evaluation will be essential components of success [25].

Ultimately, AI should not be viewed as a standalone technological solution, but as a tool that, when thoughtfully integrated, complements and augments human expertise. Its true potential will be realized only when it seamlessly fits into the complex ecosystem of pediatric rare disease care. This will empower clinicians to deliver faster, more accurate, and more reliable diagnoses for children and families navigating their challenging diagnostic journeys [43].

### 3.6. Ethical Considerations in Pediatric Settings

The integration of AI into pediatric rare disease diagnostics introduces a constellation of unique ethical and interpretational challenges, extending far beyond questions of technical performance and diagnostic accuracy. These challenges are deeply rooted in the inherent vulnerability of pediatric patients, the psychological and emotional complexities of parental decision-making, and the long-term implications that early-life genetic diagnoses may hold for children as they grow into adulthood (Table 6) [41].

One central concern is the “black-box” nature of many AI systems. While some platforms provide traceable reasoning pathways, confidence scores, or ranked output explanations that allow clinicians to understand how a conclusion was reached, many advanced deep learning models operate with undisclosed algorithms. The internal logic of their decision-making processes is often inaccessible even to developers. This lack of transparency raises fundamental questions of accountability. When an AI system suggests a diagnosis that carries life-altering consequences, whether in terms of treatment decisions, reproductive planning, or psychosocial impact, clinicians and families must face recommendations that may be difficult to interpret or contest [50].

This issue is further magnified in pediatric settings, where decisions are made not for oneself but on behalf of a child. Here, diagnostic labels extend beyond immediate medical management—they shape family identity, influence educational and social opportunities, and carry the potential for future discrimination. The introduction of AI into this delicate landscape adds another layer of interpretational difficulties. Clinicians may feel ethically conflicted about relying on algorithmic outputs they cannot fully explain, while parents may struggle to trust diagnoses perceived as generated by an impersonal machine rather than guided by human expertise and empathy [54].

The interpretation of uncertain or incidental findings becomes especially ethically charged in the pediatric context. AI-driven variant prioritization tools often highlight genes of uncertain clinical significance (VUSs) or reveal associations with adult-onset conditions unrelated to the current diagnostic question. Deciding what to report, how to communicate these findings to families, and whether any clinical action is warranted demands a careful balance between thoroughness and the potential for harm through overdiagnosis or undue anxiety [12].

Moreover, informed consent processes must evolve to encompass these complexities. Parents consenting to genomic testing for their child should be made aware not only of the nature and scope of the sequencing itself but also of the role AI algorithms play in interpreting these vast datasets. Explaining the limitations, uncertainties, and possible unintended findings of AI-assisted analysis in an accessible, non-technical manner is essential to maintaining transparency and trust [54].

Another critical consideration is equity and bias within AI systems. Most AI algorithms are trained on datasets derived from populations with disproportionate representation of certain ethnicities, geographic regions, and socioeconomic groups. As a result, diagnostic accuracy may be lower in underrepresented populations, including ethnic minorities, children from low-income regions, or those with atypical phenotypic features. In the field of rare diseases, where each case is precious and delays in diagnosis can carry lifelong consequences, such biases risk exacerbating existing disparities in healthcare access, quality, and outcomes [55].

Model performance is sensitive to population representativeness: under-sampling of ethnic and geographic groups can degrade accuracy and systematically shift errors, reinforcing inequities. Evidence from widely used clinical algorithms shows how label choices and data provenance can encode structural disparities, producing biased outputs even for an identical risk score [18]. Mitigation requires deliberate diversification of training cohorts, continuous fairness auditing, and governance frameworks that explicitly foreground equity in pediatric data use (e.g., WHO Ethics & Governance guidance; ISO/IEC 23894 AI risk-management) [56].

Furthermore, the longitudinal implications of early AI-assisted diagnoses demand careful ethical reflection. Labeling a child with a rare genetic disorder based on an AI-informed diagnosis can influence their entire medical trajectory, shape self-perception, and affect insurability or future reproductive decisions. As AI becomes increasingly integrated into newborn screening, early developmental assessments, and routine diagnostic decision-making, these implications extend from the individual child to familial and societal levels [54].

The potential psychological impact on families should not be underestimated. Receiving a diagnosis through AI-assisted processes may feel depersonalized or disempowering if not communicated within a framework of compassionate clinical care. It is critical that AI never becomes a substitute for the human connection, empathy, and meaningful conversation that families navigating rare disease diagnoses urgently need [1].

Finally, there is a growing recognition that ethical frameworks must keep pace with technological innovation. Developing guidelines and safeguards to ensure responsible, equitable, and child-centered use of AI will require broad collaboration. Clinicians, bioethicists, AI developers, policymakers, and data scientists must all work together to create standards for transparency and accountability. Equally essential is the engagement of patient advocacy groups, parents, and young people themselves in shaping these frameworks. Their experiences and perspectives provide invaluable insights into how AI can best serve patients, families, and society [54,57].

Ultimately, grounding AI innovation in ethical reflection should not be viewed as a peripheral consideration but rather as an essential component in realizing its promise. Addressing the associated ethical, interpretational, and societal challenges with foresight, humility, and inclusivity may help AI move closer to fulfilling its potential in personalized pediatric medicine [58].

### 3.7. Future Directions

Looking ahead, the path from identifying challenges to deploying AI responsibly in pediatric rare disease diagnostics hinges on targeted strategies grounded in ethical and technical rigor. For instance, improving data interoperability and tackling fragmentation will likely require not only harmonized digital standards but also the creation of shared phenotype–genotype registries across institutions [59].

Equally critical is the move toward more interpretable models. In pediatrics, the adoption of explainable AI (XAI) frameworks—paired with the involvement of pediatricians in model evaluation—can help demystify algorithmic outputs and foster clinician trust [60].

To mitigate biases inherent in AI training datasets, it is essential to diversify cohort representation and integrate continuous fairness audits. Complementing this, the ACCEPT-AI framework offers concrete ethical guidance to ensure that pediatric data usage in AI research remains equitable and child-centric [59].

Moreover, clinician uncertainty toward AI may be alleviated through embedded training, demonstration projects, and seamless integration of AI into existing workflows rather than siloed tools. Finally, advancing legal clarity and establishing frameworks for consent, liability, and data protection will anchor these innovations in governance and trust [61].

Several authors have also emphasized that the effective deployment of AI in healthcare requires not only technical advances but also policy frameworks, ethical governance, and real-world demonstration projects to ensure sustainability and trustworthiness [62].

## 4. Limitations of This Study

This review has several limitations that should be considered when interpreting its content. The field of artificial intelligence in pediatric rare disease diagnostics is advancing rapidly, and much of the available evidence remains exploratory or derived from single-center initiatives. As a result, the findings discussed here may not necessarily reflect broader or long-term clinical practice. In addition, this review is based primarily on published studies, which may be subject to reporting and publication biases, particularly with respect to positive results. Comparative evaluation across studies was not feasible due to heterogeneity in methods, datasets, and outcome measures. Moreover, we did not attempt a formal meta-analysis, and so the conclusions should be regarded as narrative and descriptive rather than quantitative. Finally, given the fast pace of model development, some examples cited may already be evolving, and their relevance may change as newer systems are validated and integrated into clinical care.

## 5. Conclusions

This narrative review synthesized current applications of AI in pediatric rare disease diagnostics, covering variant prioritization, phenotype–genotype integration, large language models, and the role of real-world data. Comparative evidence from both structured studies and clinical practice suggests that AI can complement human expertise, offering particular value in accelerating diagnoses and expanding access to specialist-level interpretation.

Throughout this review, we have highlighted the expanding role of AI in interpreting complex genomic data, aligning diverse and sometimes subtle phenotypic presentations with known genetic conditions, and supporting earlier, more personalized clinical decision-making (Table 7).

However, performance varies by disease complexity, data quality, and workflow integration. Ethical and implementation challenges, including transparency, bias, regulatory uncertainty, and clinician adoption, remain central issues that require attention to ensure equitable and trustworthy use [41,53,54].

While AI holds significant potential to reduce diagnostic delays and enable more personalized care for children with rare conditions, its impact ultimately depends on multidisciplinary collaboration, rigorous validation, and robust governance frameworks aligned with clinical realities [54].

## Figures and Tables

**Table 1:** Applications of AI in genomic data interpretation for pediatric rare diseases.

Application Area	Description	Example Tools/Platforms
Variant Prioritization	Automated ranking of genetic variants based on pathogenicity predictions, allele frequencies, and gene–disease associations	MOON (Diploid), Fabric Genomics, Emedgene, GEM
Phenotype–Genotype Matching	Linking patients’ phenotypic features (HPO terms) to known gene–disease relationships	Phenomizer, GEM
Reverse Phenotyping	AI-driven re-evaluation of clinical features based on unexpected or novel genetic findings	LLM-assisted reverse phenotyping workflows
Natural Language Processing	Extracting structured phenotypic information from unstructured clinical notes	NLP modules integrated within genomic AI pipelines
Clinical Summarization and Decision Support	Generating diagnostic hypotheses and literature-informed interpretations	ChatGPT (GPT 4.5, OpenAI), DeepSeek Medical AI

Note: This table summarizes the main application areas of AI in genomic data interpretation for pediatric rare diseases, highlighting representative tools that exemplify the range of functions—from variant prioritization to natural language processing and clinical decision support.

**Table 2:** Leading AI tools for phenotype–genotype integration in pediatric genomics.

Tool	Function	Integration	Validation Status
MOON	Variant prioritization based on the phenotype–genotype correlation	Standalone; requires manual HPO input	Used in clinical diagnostics; validated in internal benchmarking
GEM	AI-based interpretation and scoring of variants	Integrated with the Fabric Genomics platform	Deployed in hospital settings; comparative benchmarking with human panels
Phenomizer	Suggests differential diagnoses from HPO terms	Standalone; research use	Open-access tool; used in academic projects
Face2Gene	Image-based facial phenotype recognition	Mobile/web platform	High accuracy in syndromic conditions; not validated for nonsyndromic cases
Emedgene	AI-supported variant analysis with automated reporting	Commercial clinical platform	Cleared by regulatory agencies in some jurisdictions; limited open-access data
DeepPhen	Phenotype-driven gene ranking using ML	Research use; experimental	Experimental validation in selected cohorts

Note: The tools listed vary in terms of accessibility, integration into clinical pipelines, and robustness of validation. The selection is based on the recent literature and institutional experience in pediatric genomics.

**Table 3:** Comparison of AI approaches: phenotype-driven algorithms vs. large language models.

Feature	Phenotype-Driven Algorithms	Large Language Models (LLMs)
Primary Input Type	Structured data (HPO terms)	Natural language, unstructured text
Strengths	Precise gene–disease matching, standardized outputs	Flexible interpretation, literature summarization, clinical reasoning
Limitations	Dependence on structured inputs, limited in ambiguous cases	Potential hallucinations, interpretability concerns
Examples	MOON, GEM, Phenomizer	ChatGPT, DeepSeek Medical AI
Ideal Use Case	Variant prioritization with detailed phenotypic data	Complex differential diagnosis, summarizing patient histories

Note: This table summarizes key distinctions between phenotype-driven algorithms and LLMs with regard to input type, strengths, limitations, and ideal use cases in rare disease diagnostics.

**Table 4:** Algorithmic and diagnostic barriers in AI performance.

Challenge	Category	Impact on Diagnosis	Addressable by:
Unstructured EMR data	Data issue	Limits phenotypic precision; weakens AI inputs	NLP tools; structured phenotyping
Lack of interoperability	Data/workflow	Prevents integration with AI tools and databases	Cross-platform EMR integration
Clinician skepticism and unfamiliarity	Workflow/human factor	Delays adoption; mistrust of AI recommendations	Targeted training, demonstration studies
Hallucination risk in LLMs	Algorithmic/technical	Produces plausible but false diagnoses	Validation, hybrid expert oversight
Regulatory ambiguity	Legal/ethical	Unclear liability; discourages clinical use	Guidelines, legal frameworks
Bias in training data	Ethical/data quality	Overlooks underrepresented populations	Diverse datasets, fairness auditing

Note: This table outlines key challenges affecting the use of AI in diagnostics, categorized by their nature and corresponding impact on clinical utility. It also highlights potential strategies or tools that can help mitigate each issue.

**Table 5:** Clinical implementation challenges and system-level barriers.

Challenge	Description	Potential Solutions
Data Interoperability	Lack of standardized EMR ^1^ and genomic data integration	Harmonized data standards
Workflow Integration	AI tools functioning as standalone systems	Seamless integration into hospital information systems
Clinician Training and Trust	Limited familiarity with AI methodologies	Targeted educational programs— demonstration projects
Validation and Regulation	Lack of universal validation standards	Development of regulatory frameworks specific to AI diagnostics
Resource Constraints	Infrastructure and cost barriers in low-resource settings	Cloud-based AI platforms, tiered implementation models

^1^ Electronic Medical Record. Note: This table summarizes key barriers to the clinical implementation of AI tools, including technical, organizational, and regulatory challenges. It also presents potential solutions aimed at improving integration, usability, and trust in real-world healthcare settings.

**Table 6:** Ethical considerations in AI-assisted pediatric rare disease diagnostics.

Ethical Domain	Key Issues	Proposed Mitigations
Transparency and Explainability	“Black box” decision-making processes	Develop interpretable AI models, provide output rationales
Informed Consent	Complexity of explaining AI involvement to parents	Tailored consent forms detailing AI’s role, benefits, and limitations
Equity and Bias	Underrepresentation of certain ethnic groups in training datasets	Diversify training data, continuous model revalidation
Privacy and Data Security	Handling identifiable genomic and phenotypic data	Robust encryption, compliance with pediatric data protection laws
Psychosocial Impact	Emotional burden of AI-generated diagnoses	Ensure clinician-led communication with empathy and support

Note: This table highlights core ethical concerns in applying AI to pediatric rare disease diagnostics, focusing on transparency, consent, bias, privacy, and psychosocial impacts. Suggested mitigations emphasize the need for human-centered, secure, and equitable implementation.

**Table 7:** Key advantages and limitations of AI in pediatric rare disease diagnostics.

Aspect	Advantages of the AI Approach	Limitations of the AI Approach
Speed	Rapid analysis of large-scale genomic and phenotypic datasets	Limited validation for ultra-rare and atypical cases
Accuracy	High precision for syndromically well-defined conditions (e.g., achondroplasia)	Reduced accuracy in genetically heterogeneous or phenotypically ambiguous disorders
Accessibility	Expands the diagnostic capacity in settings lacking subspecialty expertise	Dependent on data quality and input standardization
Result Interpretability	Transparent algorithms in some platforms allow reasoning review	“Black box” models hinder interpretability and trust
Cost-effectiveness	Long-term reduction in diagnostic odyssey costs	Initial investment required for infrastructure and training
Ethical Considerations	Enables faster diagnosis and personalized therapies	Risks of bias propagation and unequal diagnostic accuracy across populations

Note: This table contrasts key aspects of AI-assisted diagnostics in rare diseases, outlining both the benefits and limitations across domains such as speed, accuracy, accessibility, interpretability, cost-effectiveness, and ethics.

## Data Availability

No new data were created or analyzed in this study.

## Abbreviations

The following abbreviations are used in this manuscript:

AI	Artificial Intelligence
LLM/LLMs	Large Language Model(s)
NGS	Next-Generation Sequencing
WES	Whole-Exome Sequencing
WGS	Whole-Genome Sequencing
HPO	Human Phenotype Ontology
RF	Reverse Phenotyping
VUS	Variant of Uncertain Significance
NLP	Natural Language Processing
EMR/EMRs	Electronic Medical Record(s)
EHR/EHRs	Electronic Health Record(s)
RWD	Real-World Data
RCT/RCTs	Randomized Controlled Trial(s)
OMIM	Online Mendelian Inheritance in Man
